# From Magnetic Fibers to Paper Architectures: A Lightweight Strategy for Robust Low‐Frequency Microwave Absorption

**DOI:** 10.1002/advs.77278

**Published:** 2026-08-20

**Authors:** You Wu, Yanlong Li, Haitong Sun, Tao Liu, Kehai Liu, Kaihui Liu, Zhaoxia Sun, Wenbo Ju

**Affiliations:** ^1^ School of Physics and Optoelectronics South China University of Technology Guangzhou China; ^2^ Songshan Lake Materials Laboratory Dongguan China; ^3^ Tongling Weiyi New Material Co., Ltd. Tongling China; ^4^ State Key Laboratory of Artificial Microstructure and Mesoscopic Physics School of Physics Peking University Beijing China

**Keywords:** electrodeposition, low‐frequency microwave absorption, magnetic aramid fiber, magnetic paper, Invar‐type Fe_0.64_Ni_0.36_ alloy

## Abstract

Efficient low‐frequency microwave absorption is a long‐standing challenge: carbonaceous absorbers demand bulky, centimeter‐scale thicknesses, while conventional magnetic absorbers rely on high loadings of dense magnetic filler‐ making both impractical for compact, weight‐sensitive systems. Here, we integrate the magnetic material directly into the structural fiber rather than dispersing it as filler: each filament is metallized with a dense, micrometer‐thick Fe_0.64_Ni_0.36_ (Invar‐type) alloy coating through a scalable, continuous process, and the functionalized fibers are assembled directly into paper by wet‐laid papermaking. The resulting fibers form a hierarchical conductive network that couples strong dielectric dissipation with the permeability needed to relieve the impedance‐mismatch bottleneck of low‐frequency absorbers, while preserving the mechanical properties of neat aramid paper. The paper with the optimized composition achieves effective S‐band absorption at a magnetic loading of only 21.3 wt.%. Free‐space reflectance measurements confirm a reflection loss of −21.8 dB and an effective absorption bandwidth of 1.0 GHz at a thickness of 3.3 mm. By uniting scalable fiber metallization with established papermaking, this work opens a practical route to thin, lightweight, low‐frequency absorbers and, more broadly, to multifunctional fiber‐based materials.

## Graphical abstract

A scalable process converts para‐aramid fibers into soft‐magnetic building blocks via micrometer‐thick Fe_0.64_Ni_0.36_ (Invar‐type) coatings. Assembled into paper, they form a hierarchical conductive network whose tunable permeability enables thin, lightweight microwave absorption in the low‐frequency (S‐band) regime. Beyond this, the strategy opens a route to a new class of multifunctional fiber‐based materials.

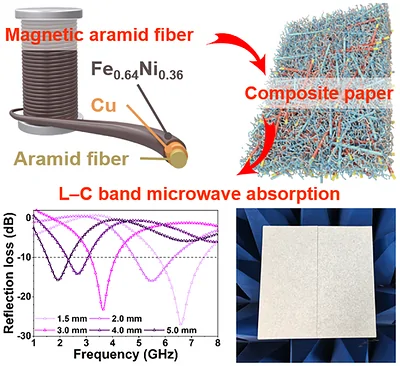

## 1 Introduction

Beyond their historical roots in military applications, microwave absorbers (MWAs) have evolved into versatile platforms for microwave manipulation, addressing the increasingly complex demands of the modern information age [1]. The rapid proliferation of fifth‐generation (5G) communication technology within the FR1 band (0.41‒7.125 GHz) [2] has intensified electromagnetic interference and pollution [3, 4, 5], while the enduring reliance on long‐range radar for detection has driven demand for advanced MWAs capable of reducing the radar cross‐section (RCS) across the L‐, S‐, and C‐bands (1–8 GHz) [6]. Converging on the same low‐frequency regime, these civilian and defense imperatives have established microwave control at these frequencies as a pivotal frontier in materials science [7]. Yet the development of low‐frequency MWAs remains fundamentally constrained by the quarter‐wavelength matching condition [8, 9], which imposes a severe thickness‐and‐weight penalty that impedes their integration into compact, weight‐sensitive systems.

The design of high‐performance MWAs necessitates the precise tuning of complex permittivity (*ε*_r_ *= ε′* ‒ *j ε″*) and permeability (*µ*_r_ *= µ′* ‒ *j µ″*) to simultaneously satisfy impedance matching at the air‐absorber interface and maximize energy dissipation within the material [1, 10]. While carbonaceous fillers‒such as carbon black [11], graphene [12], and carbon nanotubes (CNTs) [13]‒offer excellent dielectric loss and lightweight characteristics for high‐frequency (8‒18 GHz) absorption, their negligible permeability cannot compensate for their high permittivity, resulting in a severe impedance mismatch at lower frequencies. Magnetic materials present a viable solution for the low‐frequency regime, since their tunable permeability provides an additional degree of freedom to satisfy the impedance matching condition [Z = (*µ*_r_ / *ε*_r_)^1/2^ ≈ 1] [14]. Their integration, however, is impeded by high intrinsic densities (4‒8 g∙cm^−3^) [15, 16], excessive loading requirements (typically >50 wt.%) [17, 18, 19], and susceptibility to oxidation [20] and agglomeration [21].

Paper‐based architectures, defined as quasi‐two‐dimensional networks derived from stochastic fiber‐fibril assemblies [22], offer an adaptable template for addressing these multi‐variable challenges. The processing flexibility inherent to papermaking enables the construction of hierarchical conductive networks, in which high‐aspect‐ratio fillers (e.g., carbon fibers [23] and CNT‐coated fibrils [24]) lower the percolation threshold, thereby enhancing dissipative capability at minimal mass loading [23, 24, 25]. To date, paper‐based absorbers have been predominantly carbonaceous and applied mainly in the X‐band and above [24, 26], with only rare extension to the low‐frequency regime. Effective absorption in the S‐band is particularly challenging, typically demanding Jaumann [27, 28] or honeycomb [23, 29] configurations to achieve both impedance matching and strong attenuation−driving the overall thickness beyond 10 mm. Extending this paper‐based strategy to magnetic absorbers alleviates the density constraint through the intrinsic porosity of the paper, while the mechanical rigidity of the fibrous network suppresses the agglomeration of magnetic fillers. The added permeability further relaxes the impedance‐matching constraint, enabling strong low‐frequency absorption at reduced thickness.

Herein, we report a scalable, multi‐stage protocol for engineering micrometer‐thick Fe_0.64_Ni_0.36_ (Invar‐type) alloy coatings onto para‐aramid fibers via autocatalytic Cu seeding and galvanostatic electrodeposition. These functionalized fibers expand the palette of building blocks available for papermaking. In contrast to the conventional approach of dispersing discrete magnetic particles within a matrix, this strategy converts the load‐bearing fiber itself into a continuous soft‐magnetic element. Integrating these functionalized fibers into a wet‐laid paper architecture yields a hierarchical conductive network that amplifies dielectric loss while preserving the structural integrity and mechanical benchmarks of conventional aramid paper. Critically, the continuous soft‐magnetic alloy phase introduces the tunable permeability needed to overcome the impedance‐mismatch bottleneck that constrains low‐frequency MWAs. Validated by free‐space reflectance measurements, the composite papers exhibit robust S‐band absorption, achieving a maximum reflection loss (*RL*_max_) of ‒21.8 dB and an effective absorption bandwidth (*EAB*) of 1.0 GHz at a thickness of 3.3 mm. This work establishes an industrially viable foundation for a new class of compact, lightweight MWAs tailored to low‐frequency operating scenarios.

## 2 Results and Discussion

### 2.1 Magnetic Aramid Fibers

**FIGURE 1:**
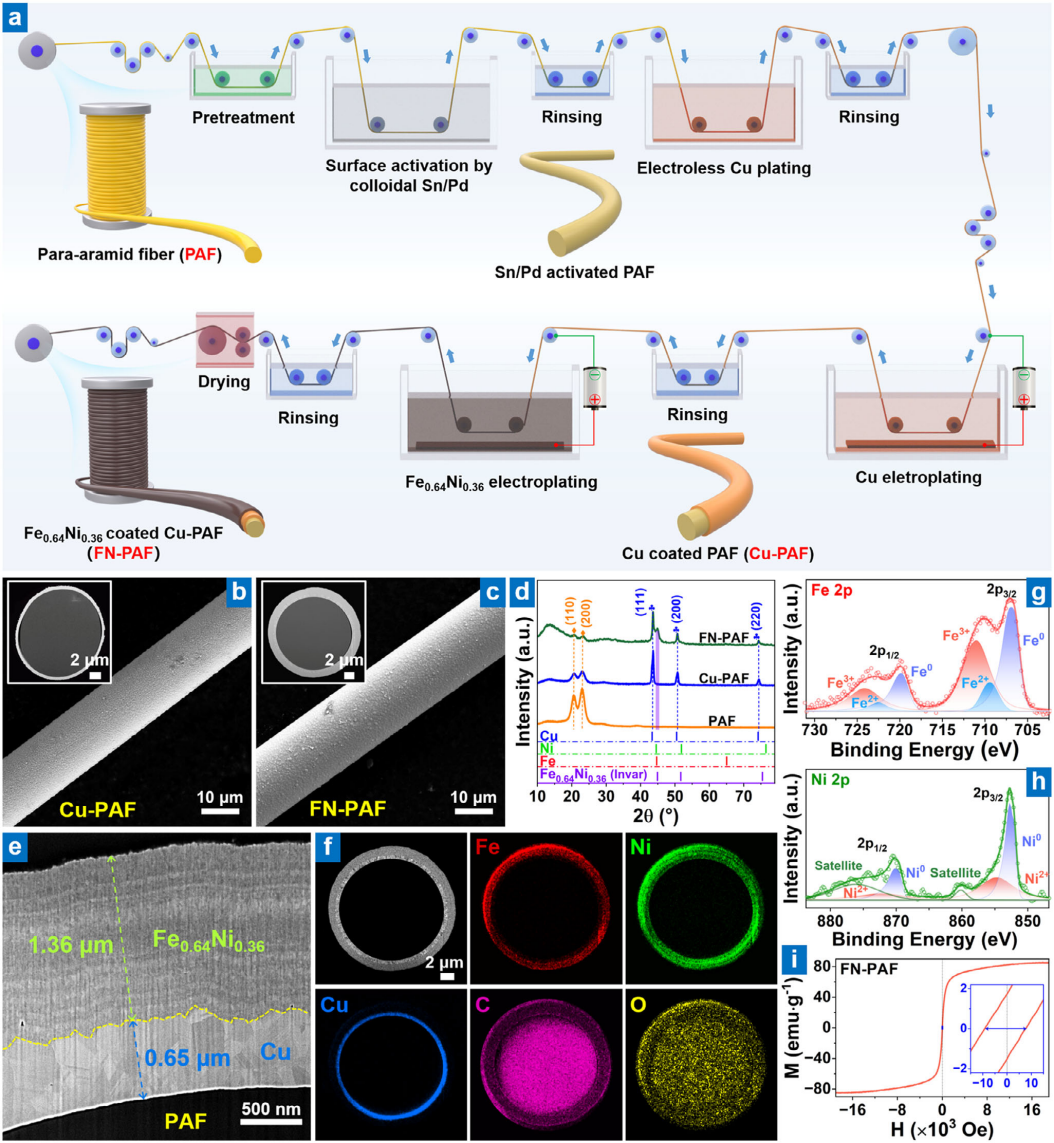
(a) Schematic of the fabrication route for the FN‐PAFs via electroless plating and electrodeposition. SEM images of (b) Cu‐PAF and (c) FN‐PAF, with insets showing the corresponding fiber cross‐sections. (d) XRD patterns of the pristine PAF, Cu‐PAF, and FN‐PAF. (e) High‐magnification SEM image of the coating cross‐section. (f) EDS elemental mapping of the FN‐PAF cross‐section. High‐resolution XPS spectra of (g) Fe 2p and (h) Ni 2p regions. (i) Magnetic hysteresis loop of FN‐PAF, with the inset showing the magnified region near the origin.

The core strategy is to convert the aramid fiber into a magnetic building block: individual filaments are metallized so that magnetic functionality resides within the fiber network itself rather than in dispersed particles, enabling magnetic aramid papers to be assembled directly from the functionalized fibers. Realizing this requires a magnetic phase that combines high magnetization with environmental durability. Among the ferromagnetic metals (Fe, Co, and Ni), the saturation magnetization (*M*_s_) decreases in the order Fe > Co > Ni [30, 31], whereas the corrosion resistance follows the opposite trend [32]. Since no single element satisfies both criteria, an Invar‐type alloy (Fe_0.64_Ni_0.36_) was selected, in which the high Fe content secures an elevated *M*_s_ while the Ni content ensures long‐term chemical durability [33]. The fabrication route (Figure 1a) converts a bare para‐aramid fiber (PAF) into a surface‐metallized fiber through a continuous sequence of surface‐engineering stages, progressing from interfacial anchoring to controlled alloy deposition. The PAFs were first acid‐etched to hydrolyze surface amide bonds [34], introducing polar carboxyl and amine moieties that promote micromechanical anchoring of the metallic layer. These etched PAFs were then sensitized in a stannous chloride (SnCl_2_) solution and activated in a palladium chloride (PdCl_2_) solution, generating metallic Pd nanoparticles that catalyze the electroless deposition of a Cu seed layer. This underlayer was subsequently thickened to over 500 nm by galvanostatic electroplating, yielding a low‐resistivity Cu‐coated PAF (Cu‐PAF) template. This high conductivity ensured a uniform current distribution during the subsequent electrodeposition of the Fe_0.64_Ni_0.36_ alloy onto the Cu‐PAF cathode. In this step, bath additives suppressed the anomalous codeposition characteristic of the Fe‒Ni system [35], thereby ensuring both stoichiometric accuracy and coating continuity. Finally, the resulting Fe_0.64_Ni_0.36_‐coated Cu‐PAFs (FN‐PAFs) were passivated with benzotriazole to form a chemisorbed barrier against oxidative degradation [36], preserving the intrinsic magnetic performance and long‐term structural integrity of the alloy coating. To demonstrate scalability, the process was implemented in a continuous filament‐winding system that coated 13 fiber bundles in parallel at a throughput of 1 m∙min^−1^ (Figure S1).

The pristine PAF has a uniform diameter of ∼ 17 µm (Figure S2). Upon Cu electrodeposition, a conformal, continuous underlayer forms around each fiber (Figure 1b), onto which the Fe_0.64_Ni_0.36_ magnetic layer is subsequently deposited. The complete multilayer coating‐ comprising the Pd catalytic seed, the Cu underlayer, and the Fe_0.64_Ni_0.36_ magnetic layer‐ reaches a total thickness of ∼ 2 µm, with a dense, pore‐free morphology and an intimate metal‐polymer interface showing no discernible delamination (Figure 1c). The FN‐PAF has a density of 3.88 g∙cm^‒3^, a 2.7‐fold value relative to the pristine substrate. X‐ray diffraction (XRD) patterns (Figure 1d) trace the evolution of the fiber through successive deposition stages: from the characteristic PAF reflections at 20.6° and 23.0° [(110) and (200) planes] [37], to Cu peaks at 43.6° and 50.7° [(111) and (200) planes], and finally to a broadened reflection at 44.9°. This feature is assigned primarily to the (111) plane of the face‐centered cubic (fcc) γ‐FeNi Invar phase [38], though it likely encompasses a range of Fe‒Ni solid solutions and elemental phases consistent with the microstructural heterogeneity inherent to high‐throughput electrodeposition.

The underlayer comprises ∼ 0.65 µm of Cu, while the Fe_0.64_Ni_0.36_ alloy layer measures ∼ 1.36 µm (Figure 1e). Notably, both dimensions remain below the calculated skin depth (δ) of these materials over the 1‒18 GHz frequency range, indicating that electromagnetic interactions can engage the full coating volume (Figure S3) [39]. Energy dispersive x‐ray spectroscopy (EDS) mapping reveals that Fe and Ni encapsulate the underlying Cu (Figure 1f). EDS line‐scan analysis gives an average Fe:Ni atomic ratio of 64:36, about which the composition varies as a gradient−Ni‐enriched at the inner interface and increasingly Fe‐rich toward the outer surface (Figure S4). The gradient reflects the anomalous codeposition of Fe−Ni, in which the alloy composition is governed by conditions at the electrode/electrolyte interface rather than by the bulk thermodynamics [40]. Hydrogen evolution during metal deposition raises the local pH, promoting Fe^2+^ hydrolysis and the formation of adsorbed Fe species that enhance Fe reduction while inhibiting Ni deposition. This resulting Fe enrichment toward the outer surface is corroborated by x‐ray photoelectron spectroscopy (XPS), which gives a surface Fe:Ni atomic ratio of 77:23 (Figure S5). High‐resolution Fe 2p and Ni 2p XPS spectra reveal the coexistence of metallic and oxidized states at the surface (Figure 1g,h). The potentiodynamic polarization curve of FN‐PAF gives a corrosion potential of −0.140 V (vs. the standard hydrogen electrode, SHE; Figure S6), lying between those of electroplated Ni (+0.069 V) [41] and Fe (−0.345 V) [42]. Although incorporating the less‐noble Fe shifts the corrosion potential negative relative to Ni, the Ni content keeps it substantially more positive than that of pure Fe, imparting adequate corrosion resistance to the Fe_0.64_Ni_0.36_ alloy and rendering the FN‐PAF sufficiently durable for wet‐laid papermaking.

Magnetic characterization reveals that the Fe_0.64_Ni_0.36_ alloy coating renders the FN‐PAF ferromagnetic, with a high *M*_s_ of 84.4 emu·g^‒1^ (Figure 1i). The hysteresis loop exhibits a low coercivity (*H*_c_) of 8.4 Oe and a small remnant magnetization (*M*_r_) of 1.76 emu·g^‒1^, indicative of negligible hysteresis loss. These soft‐magnetic characteristics allow the FN‐PAF to retain appreciable permeability into the GHz range, which is essential for magnetic MWAs [43]. The complex permeability of the Fe_0.64_Ni_0.36_ coating was characterized by the magnetic ring method (1−200 MHz) and the coaxial transmission‐line method (0.2−18 GHz). The ferromagnetic resonance near 100 MHz is followed by a gradual decrease in *µ′*, which approaches unity at ∼ 4 GHz (Figure S7). Below 4 GHz, *µ′* remains appreciably above unity, so the alloy coating retains a magnetic response across the L‐ and S‐bands.

### 2.2 Magnetic Aramid‐Based Papers

**FIGURE 2:**
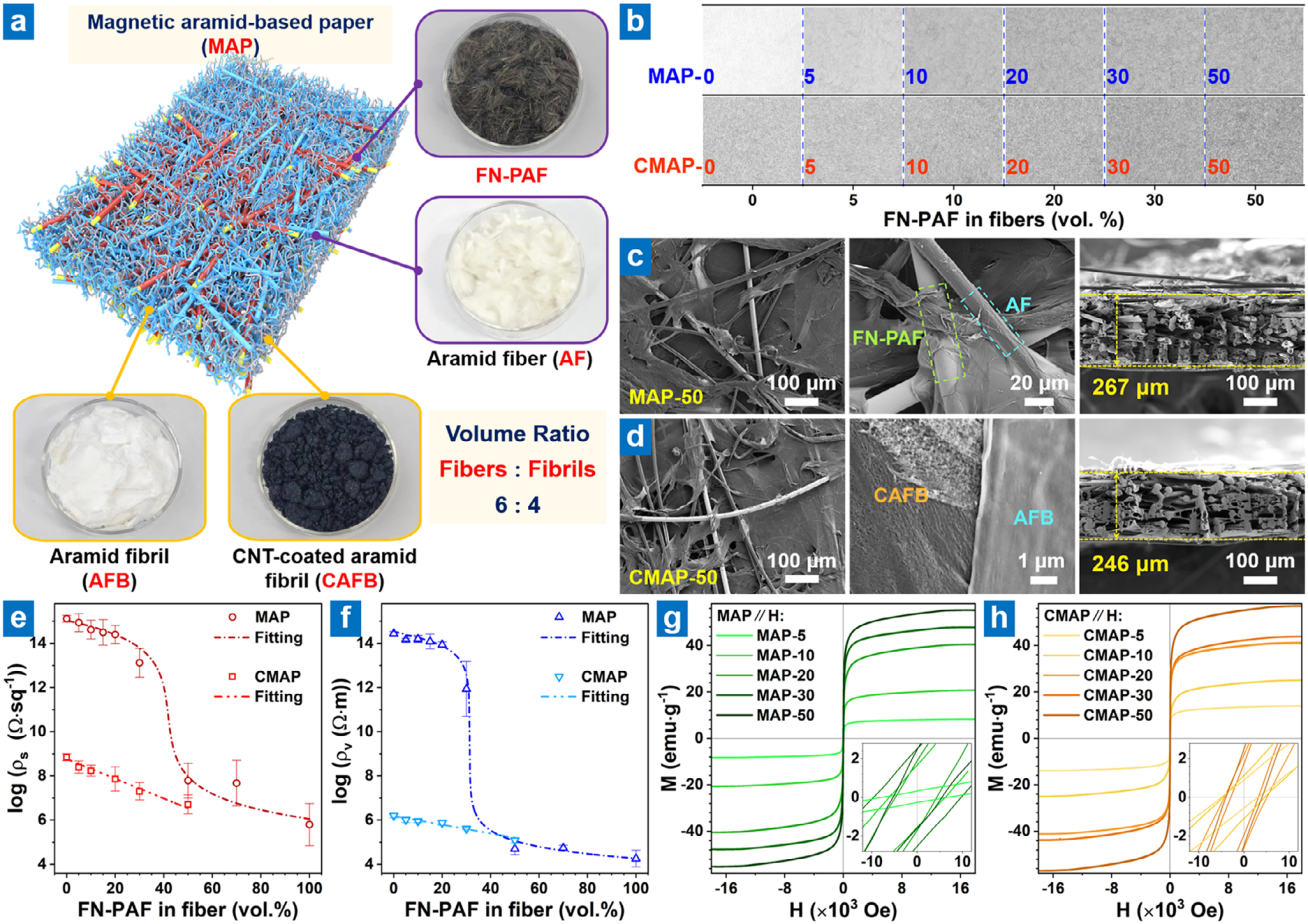
(a) Schematic of the magnetic aramid‐based papers, constructed from fibers (FN‐PAF and AF) and fibrils (AFB and CAFB) at a fixed fiber‐to‐fibril volume ratio of 6:4. Insets show photographs of the four constituents (FN‐PAF, AF, AFB, and CAFB) used for papermaking. (b) Photographs of the 12 paper types, whose designations reflect their fiber and fibril compositions. SEM images of the surface and cross‐section of (c) MAP‐50 and (d) CMAP‐50. (e) Surface and (f) bulk resistivity of the MAP and CMAP series as a function of*Φ_FN‐PAF_*, with the corresponding fitting curves. Hysteresis loops of (g) MAP and (h) CMAP with varying *Φ_FN‐PAF_*. Insets show magnified views within ±13 Oe.

Magnetic aramid‐based papers (MAPs) were assembled directly from the FN‐PAFs by wet‐laid papermaking, using short‐cut fibers (6 mm in length) and fibrils that are homogeneously distributed and randomly oriented within the sheet. Since the mechanical properties of such papers are governed by the constituent geometry and network architecture [22], a fiber‐to‐fibril volume ratio of 6:4 was maintained across all variants, yielding a nearly uniform thickness of 0.25 ± 0.02 mm. The magnetic filler loading was tuned by using FN‐PAFs either alone or blended with meta‐aramid fibers (AFs) (Figure 2a), such that the volume faction of FN‐PAFs within the total fiber content (*Φ*_FN‐PAF_) serves as the primary determinant of the electromagnetic response. The papermaking process employs two types of fibrils with contrasting electrical properties: insulating meta‐aramid fibrils (AFBs, resistivity ≈ 10^12^ Ω∙m) and conductive CNT‐coated AFBs (CAFBs, resistivity ≈ 10^5^ Ω∙m), the latter containing (4.78 ± 0.47) wt.% CNTs [24]. This seven‐order‐of‐magnitude contrast in resistivity allows the dielectric properties to be tuned via the AFB:CAFB ratio. Twelve formulations were prepared (Table S1): papers containing only AFBs are designated as MAPs, while those incorporating 20 vol.% CAFBs relative to total fibril content are designated as CNT‐incorporated MAPs (CMAPs); both series are indexed by their *Φ*_FN‐PAF_ values (Figure 2b). At a *Φ*_FN‐PAF_ of 50 vol.%, scanning electron microscopy (SEM) reveals a homogeneous dispersion of insulating and conductive fibers, which are readily distinguishable by their electron density contrast (Figure 2c). Both fiber species exhibit an isotropic, in‐plane orientation within the porous network. Fibrils adhere to the fiber surfaces or accumulate at fiber‐fiber junctions, reinforcing the network [44]. Within this architecture, the FN‐PAFs establish millimeter‐scale conductive pathways through interfacial nodal contacts, while the intermixed insulating fibers and fibrils interrupt network continuity and thereby modulate the resistivity. In the CMAP variants, the CAFB surfaces exhibit a distinctly textured morphology arising from entangled CNT networks, in contrast to the smooth topography of the AFBs (Figure 2d). These CNT layers introduce microscale conductive networks that complement the millimeter‐scale FN‐PAF pathways, yielding a hierarchical conductive architecture that spans several length scales.

The surface resistivity (*ρ*_s_) and bulk resistivity (*ρ*_v_) profiles of the MAP and CMAP series reveal contrasting charge‐transport behavior (Figure 2e,f). For the MAPs, conduction is mediated exclusively by the FN‐PAF network, exhibiting characteristic percolation behavior [45]. In the sub‐percolative regime, *ρ*_s_ remains near 10^14^ Ω∙sq^‒1^; once the critical *Φ*_FN‐PAF_ is exceeded, the formation of long‐range conductive pathways produces a multi‐order‐of‐magnitude drop in resistivity. *ρ*_v_ exhibits a similar transition, though at a lower threshold. Fitting these resistivity data as a function of *Φ*_FN‐PAF_ to the McLachlan generalized effective‐medium model [46] yields percolation thresholds (*Φ*_c_) of 41.8 vol.% for *ρ*_s_ and 31.3 vol.% for *ρ*_v_ (Table S2). These *Φ*_c_ values are pivotal for tailoring microwave absorption performance, since they mark the compositional window in which dielectric loss and impedance matching must be balanced: sufficient conductivity is required to maximize the attenuation constant (α), yet excessive conductivity in the over‐percolative regime degrades impedance matching and produces strong interfacial reflection [47, 48]. In the CMAP variants, by contrast, the CNT‐decorated fibrils provide microscale conductive networks that establish a baseline conductivity even in the absence of FN‐PAFs. CMAPs therefore exhibit lower resistivity than MAPs throughout the sub‐percolative regime. As *Φ*_FN‐PAF_ increases toward 50 vol.%, the resistivity declines only gradually, reflecting a transition from CNT‐mediated to FN‐PAF‐dominated conduction. Beyond 50 vol.%, the resistivities of the two series converge, indicating that charge transport is governed by the long‐range FN‐PAF network in both architectures.

The aramid papers become ferromagnetic upon the incorporation of FN‐PAFs, with *M*_s_ increasing with *Φ*_FN‐PAF_ from 8.3 emu∙g^‒1^ at 5 vol.% (MAP‐5) to 55.4 emu∙g^‒1^ at 50 vol.% (MAP‐50) (Figure 2g). Consistent with the soft‐magnetic nature of the Fe_0.64_Ni_0.36_ coating, all variants retain a low *H*_c_ (<10 Oe). The addition of CNTs has a negligible effect on these magnetic parameters, as the CMAP variants show hysteresis behavior nearly identical to the MAP series at equivalent *Φ*_FN‐PAF_ (Figure 2h). The FN‐PAFs also exhibit pronounced uniaxial magnetic anisotropy arising from their high aspect ratio, with the easy axis aligned along the fiber length [49]. Their preferential in‐plane orientation within the paper produces an anisotropic magnetic response, with markedly higher susceptibility parallel to the paper surface than in the transverse direction (Figure S8). The composite papers also exhibit strong mechanical performance, with an average tensile strength of ∼3.5 MPa and a mean elastic modulus of ∼0.28 GPa (Figure S9)−comparable to neat aramid paper measured under identical conditions [24]−indicating that mechanical integrity is preserved despite the electromagnetic functionalization.

### 2.3 Electromagnetic Properties

**FIGURE 3:**
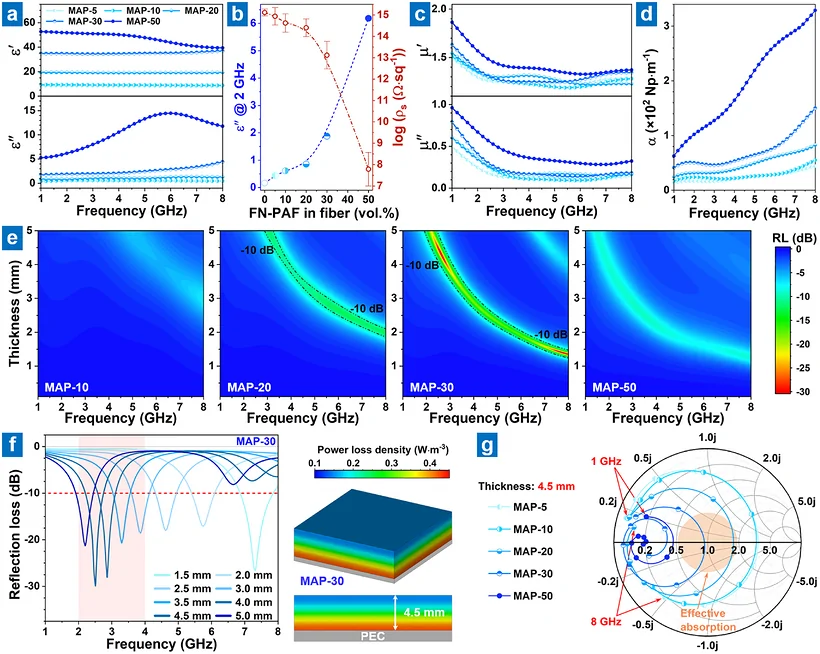
Electromagnetic properties of the MAPs over the L‒C band: (a) permittivity, (b) comparison of *ε″* at 2 GHz and *ρ*_s_ for MAPs with varying *Φ*_FN‐PAF_, (c) permeability, and (d) attenuation constant. (e) Reflection loss maps for MAP‐10, MAP‐20, MAP‐30, and MAP‐50 at thicknesses from 0.1 to 5.0 mm. (f) Reflection loss spectra of MAP‐30 at thicknesses from 1.5 to 5.0 mm, with the simulated power‐loss density distribution in a 4.5 mm MAP‐30 absorber at 2.7 GHz. (g) Smith charts of the MAPs at 4.5 mm thickness, with data points at 1.0 GHz intervals.

The complex permittivity and permeability were characterized by the coaxial transmission‐line method over 1‒18 GHz [50, 51]. To accommodate sample‐thickness constraints, measurements were performed on stacked paper sheets (Figure S10), and single‐sheet parameters were extracted by thickness normalization. Neat aramid paper exhibits *ε′* ≈ 1.2 with negligible *ε″*, owing to the low polarizability of the aramid and the air‐filled interstices (Figure S11) [24, 52]. Introducing 5 vol.% FN‐PAFs (MAP‐5) raises *ε′* above 8.0 across the L‒C band and initiates dielectric loss, with *ε″* reaching ∼0.44 (Figure 3a). *ε′* increases with *Φ*_FN‐PAF_, particularly in the low‐frequency regime where antenna‐resonance artifacts are minimized (Figure S12a). *ε″* remains low at small *Φ*_FN‐PAF_ but rises abruptly once the *Φ*_c_ is exceeded (Figure 3b). Below *Φ*_c_, isolated FN‐PAFs contribute limited conduction loss, yielding modest *ε″*; above *Φ*_c_, continuous conductive pathways form, producing a pronounced increase in *ε″* that mirrors the resistivity drop and identifies conduction loss as the primary dielectric dissipation mechanism.

The MAP series retains permeability into the GHz range, with *µ′* increasing with *Φ*_FN‐PAF_ from 1.30 (MAP‐5) to 1.57 (MAP‐50) at 2 GHz (Figure 3c). The decay of *µ′* and *µ″* between 1 and 4 GHz reflects the high‐frequency tail of the sub‐GHz ferromagnetic resonance observed in the FN‐PAF permeability spectrum (Figure S7). Above 4 GHz, the eddy‐current coefficient [*C*_0_ = *µ″* ∙(*µ′*)^‒2^ ∙ *f* ^‒1^] stabilizes at a frequency‐independent value (Figure S13), indicating that eddy‐current loss dominates the magnetic dissipation. Nonetheless, the magnitude of permittivity greatly exceeds that of the permeability, indicating that dielectric loss governs the overall energy dissipation. This is corroborated by the attenuation constant α, which shows a distinct percolation transition consistent with conduction‐dominated dielectric loss (Figure 3d).

The microwave absorption performance of the MAP series was evaluated using the metal‐backed single‐layer (Dallenbach) model [53], with reflection loss (*RL*) and impedance matching computed over a range of absorber thicknesses from the measured electromagnetic parameters (Figure S14). Among the MAPs, only MAP‐20 and MAP‐30 achieve effective absorption (*RL* < ‒10 dB) within the L‒C band (Figure 3e). For MAP‐30, increasing the thickness from 1.5 to 5.0 mm shifts the absorption peak from 7.3 to 2.2 GHz (Figure 3f), consistent with quarter‐wavelength behavior, in which the matching thickness is inversely proportional to frequency [54]. Optimal performance occurs at 4.5 mm, where MAP‐30 reaches an *RL*_max_ of ‒29.4 dB at 2.5 GHz with an *EAB* of 0.5 GHz. At this thickness, the simulated volumetric power‐loss density is spatially inhomogeneous, with dissipation concentrated near the conductor backing and decreasing toward the air interface (Figure S15). Smith‐chart analysis clarifies the impedance‐matching behavior underlying the absorption [55]. At 4.5 mm, the normalized input‐impedance (*Z* = *Z*_in_ / *Z*_0_) trajectories shift toward lower values as *Φ*_FN‐PAF_ increases (Figure 3g). The MAP‐20 and MAP‐30 trajectories traverse the effective‐absorption region, with MAP‐30 achieving a broader *EAB* because its trajectory spans a wider frequency range within this zone. In contrast, the MAP‐5 and MAP‐10 trajectories remain outside the region because their attenuation is insufficient, whereas the MAP‐50 trajectory bypasses it entirely owing to impedance mismatch. Together, these results underscore the need to optimize *Φ*_FN‐PAF_ to match the input impedance to free space while maintaining adequate electromagnetic loss.

**FIGURE 4:**
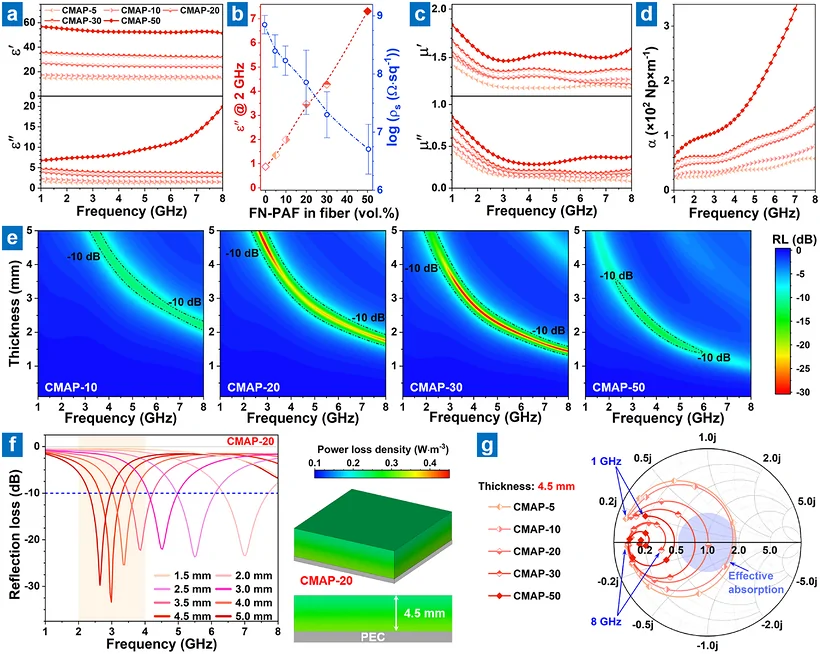
Electromagnetic properties of the CMAPs over the L‒C band: (a) permittivity, (b) comparison of *ε″* at 2 GHz and *ρ*_s_ for CMAPs with varying *Φ*_FN‐PAF_, (c) permeability, and (d) attenuation constant. (e) Reflection loss maps for CMAP‐10, CMAP‐20, CMAP‐30, and CMAP‐50 at thicknesses from 0.1 to 5.0 mm. (f) Reflection loss spectra of CMAP‐20 at thicknesses from 1.5 to 5.0 mm, with the simulated power‐loss density distribution in a 4.5 mm CMAP‐20 absorber at 2.7 GHz. (g) Smith charts of the CMAPs at 4.5 mm thickness, with data points at 1.0 GHz intervals.

The incorporation of CNTs offers a viable route to tune the electromagnetic parameters toward optimized impedance matching and sufficient attenuation. The CMAP series incorporated CAFBs at 20 vol.% of the fibril component, with an overall fiber‐to‐fibril volume ratio of 6:4. Assuming comparable densities of the fibers and fibrils, and given the (4.78 ± 0.47) wt.% CNT content of the CAFBs [24], the CNTs constitute only ∼0.4 wt.% of the entire paper. Despite this minimal loading, the microscale conductive networks formed by the CNTs markedly increase the permittivity relative to the corresponding MAPs, with *ε″* showing the most pronounced increase (Figure S16a). For example, CMAP‐5 exhibits *ε′* > 14.0 and *ε″* > 1.22 across the L‒C band, compared with *ε′* ≈ 8.0 and *ε″* ≈ 0.44 for MAP‐5 (Figure 4a). The *ε″* of the CMAP series increases quasi‐linearly with *Φ*_FN‐PAF_, accompanied by an exponential decrease in resistivity (Figure 4b), consistent with conduction loss as the primary dielectric dissipation mechanism. Interfacial polarization is also expected to contribute, particularly at the numerous heterointerfaces within the hierarchical conductive network formed by the CNTs and FN‐PAFs. The magnetic behavior is essentially unchanged by CNT incorporation: the CMAPs show permeability values and relaxation behavior comparable to the MAPs, with ferromagnetic resonance dominant below 4 GHz and eddy‐current loss prevailing at higher frequencies (Figure 4c and Figure S17). As a result, the attenuation constant α of the CMAPs consistently exceeds that of the MAPs at equivalent *Φ*_FN‐PAF_ (Figure 4d), confirming the enhanced dissipation of the hierarchical conductive architecture.

The microwave absorption performance of the CMAPs is significantly enhanced relative to the MAPs, with all investigated compositions achieving effective absorption across the L‒C band (Figure S18). Notably, CMAP‐20 shows excellent S‐band absorption with an extended *EAB*, whereas CMAP‐30 is optimized for the C‐band (Figure 4e). For CMAP‐20, the *RL* peak shifts systematically from 7.0 to 2.6 GHz as the thickness increases from 1.5 to 5.0 mm (Figure 4f). The best performance occurs at a matching thickness of 4.5 mm, where CMAP‐20 reaches an *RL*_max_ of ‒33.4 dB at 3.0 GHz with an *EAB* of 0.7 GHz. The simulated power‐loss density within CMAP‐20 is nearly homogeneous, approaching 0.3 W∙m^‒3^ throughout the volume, indicating better impedance matching than the gradient distribution observed in MAP‐30 (Figure S19). Smith‐chart analysis supports this (Figure 4g): the CMAP input‐impedance trajectories lie closer to the effective‐absorption region than those of the MAPs, and the CMAP‐20 trajectory in particular remains near the matched center across a broad frequency range, enabling the wide low‐frequency *EAB*. CMAP‐20 thus represents a promising candidate for low‐frequency MWAs, though its practical performance requires verification by experimental free‐space reflectance measurements.

### 2.4 Free‐Space Microwave Absorption Performance

**FIGURE 5:**
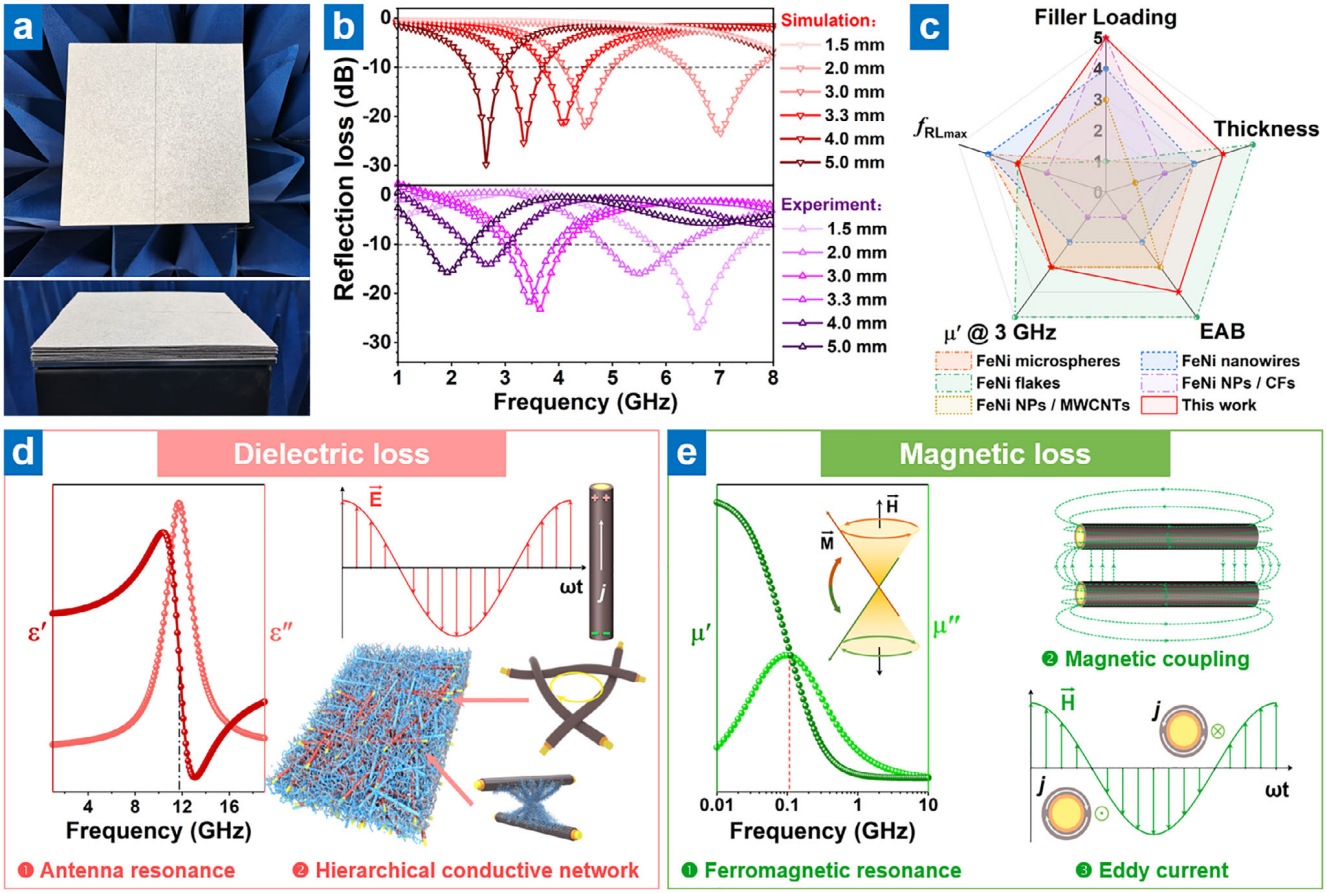
(a) Photograph of a CMAP‐20 absorber (5.0 mm) placed on a metal backing plate. (b) Simulated (upper) and experimental (lower) RL spectra of CMAP‐20 at various thicknesses. (c) Radar chart comparing the S‐band absorption performance of CMAP‐20 with five other FeNi‐based magnetic absorbers. Schematics of electromagnetic energy‐dissipation mechanisms in CMAPs: (d) dielectric loss and (e) magnetic loss.

The predicted absorption performance was experimentally validated by free‐space reflectance measurements using the NRL (Naval Research Laboratory) arch method, in which metal‐backed absorbers were illuminated at normal incidence [56]. Given the uniform single‐layer thickness of CMAP‐20 (∼0.25 mm), absorbers of different thicknesses were assembled by simple layer stacking: a 20‐layer stack, for example, yields a 5.0 mm absorber (Figure 5a). The sample dimensions (180 mm × 180 mm) were kept large compared with the operating wavelength to mitigate edge diffraction. The free‐space reflectance spectra confirm that CMAP‐20 absorbers achieve effective absorption across the L‒C band for thicknesses ≥1.5 mm (Figure S20). At 2.0 mm, the simulated *RL* spectrum predicts an absorption peak at 7.0 GHz with an *EAB* of 1.4 GHz (Figure 5b, upper panel); free‐space measurement confirmed effective absorption, with the peak at 5.5 GHz and an expanded *EAB* of 1.6 GHz (Figure 5b, lower panel). This shift is primarily attributed to interlayer interference and multiple reflections within the laminated structure, which alter the effective electromagnetic parameters relative to the homogeneous single‐layer model [57, 58]. As the stack thickness increases, the absorption peaks move to lower frequencies. At 3.3 mm, CMAP‐20 exhibits an *RL*_max_ of –21.8 dB at 3.5 GHz with an *EAB* of 1.0 GHz, demonstrating broadband S‐band absorption. At 5.0 mm, effective absorption extends into the L‐band, spanning 1.5‒2.4 GHz. The interlayer interference becomes more pronounced as the number of layers increases, producing larger frequency shifts and broader bandwidths than predicted by the single‐layer simulation. Despite these interfacial effects, the overall agreement between simulation and experiment validates CMAP‐20 as a compelling candidate for low‐frequency MWAs.

To contextualize this S‐band capability, CMAP‐20 was benchmarked against five representative FeNi‐based magnetic MWAs with diverse morphologies and composite architectures [59, 60, 61, 62, 63]. Performance was assessed across five parameters‒filler loading, matching thickness, *EAB*, *µ′* at 3 GHz, and *RL*_max_ frequency‒each normalized on a 0‒5 scale per the criteria in Table S3 and displayed as a radar chart (Figure 5c). Owing to the high aspect ratio and core‐shell morphology of the FN‐PAFs, CMAP‐20 achieves efficient attenuation at a magnetic filler loading of only 21.3 wt.% (Table S4), a substantial reduction from the ≥80 wt.% typically required by particulate or flake‐based FeNi systems (Table S5). Moreover, CMAP‐20 delivers broadband S‐band absorption (*EAB* ≈ 1 GHz) at a compact matching thickness of 3.3 mm, outperforming most reported magnetic MWAs that require greater thicknesses yet offer narrower bandwidths. This simultaneous reduction of magnetic loading and matching thickness establishes CMAP‐20 as a promising lightweight solution for S‐band applications under stringent weight and volume constraints.

Across the CMAP series, dielectric loss is the primary channel for electromagnetic energy dissipation, even though magnetic constituents are present (Figure 5d). Its dominant mechanism, however, changes with frequency. In the L–C band, dielectric loss prevails, arising from electron migration through the percolating networks formed by the FN‐PAFs and CNTs, augmented by multi‐scale interfacial polarization. Toward the X and Ku bands, antenna resonance takes over: the 6 mm FN‐PAFs act as half‐wavelength resonators (effective resonance near 12.0 GHz in the composite medium), generating intense local electric fields that enhance dissipation through the associated polarization loss‐ a response confirmed by the corresponding dispersion features in the permittivity spectra (Figures S12a and S16a). The magnetic phase contributes far less to direct dissipation, and its two loss channels vary with frequency (Figure 5e). The complex permeability spectra (0.01−1 GHz) locate the resonance peak at ∼0.09 GHz−well below the working band (Figure S21). Consequently, magnetic loss across 1−18 GHz stems only from the high‐frequency tail of this resonance and from eddy currents. Shifting this resonance to higher frequency through magnetic‐coating design could raise the in‐band permeability and strengthen the resonant contribution [64]. This gain is fundamentally bounded by Snoek's limit, which fixes the product of the permeability and the resonance frequency for a given saturation magnetization [65]; surpassing it would require additional strategies such as anisotropy engineering and morphological control [14]. The decisive contribution of the magnetic phase therefore lies not in dissipation but in impedance matching. At low frequencies in particular, its permeability counterbalances the high permittivity, easing wave penetration and suppressing front‐surface reflection, which conventional non‐magnetic absorbers cannot achieve. This is what enables effective S‐band absorption at low filler loading and compact thickness, distinguishing the CMAP design from purely dielectric systems.

## 3 Conclusions

This work establishes a strategy in which the aramid fiber itself is converted into a magnetic building block (FN‐PAF): individual filaments are metalized with a dense, micrometer‐thick Fe_0.64_Ni_0.36_ (Invar‐type) alloy coating through a scalable, continuous process. This metallization provides a general pathway for functionalizing insulating high‐performance fibers and expands the palette of building blocks available for papermaking. The wet‐laid paper architecture organizes the FN‐PAFs and CNT‐coated fibrils (CAFBs) into a hierarchical conductive network, in which the high‐aspect‐ratio FN‐PAFs provide long‐range, millimeter‐scale pathways and the CAFBs contribute microscale CNT networks. This architecture enhances dielectric dissipation while preserving the mechanical properties of neat aramid paper. Crucially, the soft‐magnetic alloy phase supplies the permeability needed to relieve the impedance‐mismatch bottleneck characteristic of low‐frequency MWAs. The experimental validated S‐band performance of CMAP‐20 (magnetic fiber loading ∼21.3 wt.%)‒an *RL*_max_ of ‒21.8 dB and an *EAB* of 1.0 GHz at a matching thickness of 3.3 mm‒demonstrates the effectiveness of this fiber‐derived magnetic strategy for weight‐ and volume‐constrained applications. Beyond this specific demonstration, these findings offer a practical foundation for compact, lightweight absorbers in the low‐frequency regime. Extending the absorption bandwidth further will require sustaining higher permeability at GHz frequencies, which may be achieved by tailoring the composition and anisotropy of the magnetic coatings to surpass Snoek's limit.

## 4 Experimental Section

Detailed information about chemicals and raw materials is provided in the Supporting Information. The Fe_0.64_Ni_0.36_ (Invar‐type) alloy‐coated para‐aramid fibers (FN‐PAFs) were synthesized via an optimized multi‐stage protocol (Section 1, SI). PAF substrates were ultrasonically pre‐treated in a hydrochloric acid (HCl) solution and rinsed in deionized water. Surface sensitization was performed in an acidic stannous chloride (SnCl_2_) solution, and activation in an acidic palladium chloride (PdCl_2_) solution. The pre‐treated fibers were continuously drawn through these colloidal baths to form a Sn/Pd layer on the fiber surface via a localized redox reaction, in which Sn(II) reduces Pd(II) to metallic Pd clusters. An electroless Cu seed layer was then deposited by autocatalytic reduction from a bath containing copper sulfate (CuSO_4_), formaldehyde, and disodium ethylenediaminetetraacetate (Na_2_EDTA). This seed layer was reinforced by galvanostatic Cu electroplating in an acidic CuSO_4_ solution containing functional additives. The resulting Cu‐coated PAFs (Cu‐PAFs) were continuously processed through a Fe_0.64_Ni_0.36_ plating bath containing ferrous sulfate (FeSO_4_), nickel sulfate (NiSO_4_), nickel chloride (NiCl_2_), and a system of additives. Finally, the FN‐PAF surface was passivated with benzotriazole.

The polyelectrolyte multilayer technique was employed to assemble single‐walled carbon nanotubes (SWCNTs) onto the surface of aramid fibril (AFB) [24]. Surface functionalization was carried out by sequential deposition of poly (diallyl dimethyl ammonium chloride) (PDDA), poly (sodium 4‐styrenesulfonate) (PSS), and PDDA. The functionalized AFBs were then immersed in an SWCNT dispersion to form a uniform CNT coating, driven by electrostatic attraction between the negatively charged carboxyl groups on the SWCNTs and the positively charged outermost PDDA layer.

The papermaking process was adapted from established protocols [23]. FN‐PAFs and aramid fibers (AFs) served as the structural fibers, while CNT‐coated AFBs (CAFBs) and AFBs acted as the fibrillar binders. A consistent fiber‐to‐fibril volume ratio of 6:4 was maintained across all variants. The volume fraction of FN‐PAFs within the total fiber content (*Φ*_FN‐PAF_) served as the primary determinant of the electromagnetic response.

The phase and surface structures of the FN‐PAFs and CAFBs were characterized by x‐ray diffraction (XRD, Bruker D8 ADVANCE diffractometer, Cu Kα radiation), x‐ray photoelectron spectroscopy (XPS, PHI GENESIS 500, Al Kα x‐ray source), and Raman spectroscopy (Renishaw InVia). Surface topography and cross‐sectional features were examined by scanning electron microscopy (SEM, Zeiss Gemini SEM 500), with elemental mapping by an integrated energy‐dispersive x‐ray spectrometer (EDS, Oxford Instruments ISIS). Radial cross‐sections of fibers were prepared by focused‐ion‐beam milling (FIB, Zeiss Crossbeam 350). Magnetic hysteresis loops were measured with a vibrating‐sample magnetometer (VSM, East Changing 3105). The linear resistance of the metallized aramid fiber bundles was measured with a digital multimeter (CHNT, ZTW0111C). Surface resistivity (ρ_s_) and bulk resistivity (ρ_v_) of the composite papers were measured with an ultra‐high‐resistance microcurrent tester (Suzhou JG, ST2643), following China National Standards GB/T 31838.2‐2019 and GB/T 31838.3‐2019. Mechanical properties were evaluated with a tensile tester (Lugong, STD1000). The corrosion behavior of FN‐PAF was assessed by potentiodynamic polarization using an electrochemical workstation (Gamry Reference 600). Complex permeability over 1‒200 MHz was measured with an impedance analyzer (Keysight E4991B), and the electromagnetic parameters over 0.1‒18 GHz with a vector network analyzer (Anritsu MS46322B). Free‐space reflectance measurements were performed by Guizhou Plato Electronics Technology Co., Ltd. using the NRL arch method.

## Author Contributions

**Kaihui Liu**: supervision, funding acquisition. **Kehai Liu**: supervision, resources. **You Wu**: methodology, data curation, investigation, formal analysis, writing – original draft, visualization. **Yanlong Li**: data curation, formal analysis, visualization. **Haitong Sun**: methodology, resources. **Tao Liu**: software, formal analysis. **Wenbo Ju**: conceptualization, methodology, validation, supervision, funding acquisition, project administration, resources, writing – review and editing, formal analysis. **Zhaoxia Sun**: methodology, investigation, supervision.

## Funding

The authors gratefully acknowledge the financial support from the National Natural Science Foundation of China, Grant No. U21A2093. Fundamental Research Funds for the Central Universities, Grant No. 2024ZYGXZR018. Introduces Innovative and Entrepreneurial Team Project of Guangdong Province, Grant No. 2021ZT09Z109.

## Conflicts of Interest

The authors declare no conflict of interest.

## Supporting information




**Supporting File**: advs77278‐sup‐0001‐SuppMat.docx.

## Data Availability

The data that support the findings of this study are available from the corresponding author upon reasonable request.
