# Supplementary material for: From Magnetic Fibers to Paper Architectures: A Lightweight Strategy for Robust Low‐Frequency Microwave Absorption
Source: Adv Sci (Weinh). 2026 Aug 20:e77278. Online ahead of print. doi: 10.1002/advs.77278 (PMC13494274; doi:10.1002/advs.77278)
Supplement: Supplementary file 1 [file ADVS-9999-e77278-s001.docx]

**From Magnetic Fibers to Paper Architectures: A Lightweight Strategy for Robust Low-Frequency Microwave Absorption**

You Wu ^1^, Yanlong Li ^2^, Haitong Sun ^3^, Tao Liu ^1^, Kehai Liu ^2^, Kaihui Liu ^2,4^, Zhaoxia Sun ^2,^*, Wenbo Ju ^1,^*

1. School of Physics and Optoelectronics, South China University of Technology, Guangzhou, 510640, China

2. Songshan Lake Materials Laboratory, Dongguan, 523808, China

3. Tongling Weiyi New Material Co., Ltd., Tongling, 244100, China

4. State Key Laboratory for Mesoscopic Physics, School of Physics, Peking University, Beijing, 100091, China

E-mail: W. Ju (wjuphy@scut.edu.cn), Z. Sun (sunzhaoxia@sslab.org.cn)

Keywords:

Magnetic aramid fiber, magnetic paper, low-frequency microwave absorption, electrodeposition, Invar-type Fe_0.64_Ni_0.36_ alloy

**Section 1. Synthesis of magnetic aramid fibers**

The Fe_0.64_Ni_0.36_ (Invar-type) alloy-coated para-aramid fibers (FN-PAFs) were synthesized via an optimized multi-stage protocol (Tongling Weiyi New Materials Co. LTD). PAF substrates were ultrasonically pre-treated (*f* = 40 kHz) in a 0.6 mol·L^‒1^ hydrochloric acid (HCl) solution for 3 minutes, then rinsed in deionized water for a 60 seconds to clean the surface. Surface sensitization was performed in an aqueous bath containing 10 g·L^‒1^ stannous chloride dihydrate (SnCl_2_·2H_2_O) and 0.5 mol·L^‒1^ HCl at 30 °C, and activation in a 0.1 g·L^‒1^ palladium chloride (PdCl_2_) solution in 1.2 mol·L^‒1^ HCl. The pre-treated fibers were continuously drawn through these colloidal bath to form a Sn/Pd layer on the fiber surface via a localized redox reaction in which Sn(II) reduces Pd(II) to metallic Pd clusters. An electroless Cu seed layer was then deposited from a bath containing 20 g·L^‒1^ copper sulfate (CuSO_4_), 33 mmol·L^‒1^ formaldehyde, and 45 g·L^‒1^ disodium ethylenediaminetetraacetate (Na_2_EDTA). After the pH was adjusted to 12.5 with NaOH and the bath pretreated to 50 °C for 30 minutes under magnetic stirring, the activated fibers were drawn through the Cu bath to form a continuous conductive underlayer by autocatalytic reduction on the Pd nucleation sites.

**Figure S1.** Photographs of (a) coated 13 fiber bundles, (b) the fiber-winding process, and (c) a coil of magnetic aramid fibers.

To achieve the target conductive profile, the electroless seed layer was reinforced by galvanostatic Cu electroplating. The bath contained 0.8 mol·L^‒1^ CuSO_4_ in deionized water acidified with 0.37 mol·L^‒1^ H_2_SO_4_, with 0.5 mol·L^‒1^ HCl added to maintain the required Cl^‒^ concentration. Additives, including brighteners and leveling agents, were incorporated to produce a dense, coherent underlayer with minimal surface defects. The resulting Cu-coated PAFs (Cu-PAFs) were then continuously processed through a Fe_0.64_Ni_0.36_ alloy plating bath containing 66 g·L^‒1^ ferrous sulfate heptahydrate (FeSO_4_·7H_2_O), 150 g·L^‒1^ nickel sulfate hexahydrate (NiSO_4_·6H_2_O), and 45 g·L^‒1^ nickel chloride hexahydrate (NiCl_2_·6H_2_O), adjusted to achieve an Fe:Ni atomic ratio of 64:36 in the coating. To regulate the anomalous codeposition and stabilize the micrometer-thin coating, the bath was supplemented with a combination of additives, including pH buffers, ionic stabilizers, and grain-refiners. Each deposition stage was followed by thorough rinsing in deionized water to remove residual electrolyte and prevent cross-contamination. The electrodeposition was performed at a current density of 3 A·dm^‒2^, using the respective metal shots in titanium baskets as anodes. Coating thickness was monitored in real time with an in-line non-contact gauge with a 30-second sampling interval. Finally, the FN-PAF surface was passivated with benzotriazole, which coordinates with surface metal atoms to form a chemisorbed polymeric film. The FN-PAFs were then dried and collected by continuous filament winding, yielding filaments with a multilayer structure comprising a ~0.6 μm Cu underlayer and a ~1.4 μm Fe_0.64_Ni_0.36_ functional coating.

**Figure S2.** SEM image of a PAF fiber.

The linear resistances of Cu-PAF and FN-PAF were measured as 7.2 Ω·m^‒1^ and 10.2 Ω·m^‒1^, respectively. From the coating dimensions, the corresponding direct-current (DC) conductivities of the Cu and Cu@Fe_0.64_Ni_0.36_ layers were calculated as 1.75 × 10^7^ S·m^‒1^ and 4.68 × 10^6^ S·m^‒1^, both below that of bulk Cu (5.96 × 10^7^ S·m^‒1^). The skin depth over 1−18 GHz was then calculated from equation S1^[1]^ :

$\delta=\sqrt{\frac{2}{\omega\mu_{0}\mu_{r}\sigma}}=\sqrt{\frac{1}{\pi f\mu_{0}\mu_{r}\sigma}}$ (S1)

where *σ* is the conductivity, *μ*_0_ and *μ*_r_ are the vacuum and relative permeability, and *ω* and *f* represent the angular frequency and frequency of electromagnetic wave, respectively. Because Cu is non-magnetic and the Fe_0.64_Ni_0.36_ layer exhibits low relative permeability (*μ′* ≈ 1−1.6) across the investigated frequency range, *μ*_r_ was set to 1 for both layers; this yields a conservative estimate of the Fe_0.64_Ni_0.36_ skin depth.

**Figure S3.** Calculated skin depths of the Cu and Cu@Fe_0.64_Ni_0.36_ layers over 1−18 GHz.

Surface topography and cross-sectional features were examined by scanning electron microscopy (SEM, Zeiss Gemini SEM 500), with elemental mapping by an integrated energy-dispersive X-ray spectrometer (EDS, Oxford Instruments ISIS). Radial cross-sections of the fibers were prepared by focused-ion-beam (FIB) milling (Zeiss Crossbeam 350).

**Figure S4.** (a) EDS spectrum and the SEM image showing the line-scan path, and (b) corresponding elemental distributions of Cu, Fe, and Ni along the path.

The surface composition of the FN-PAFs was characterized by X-ray photoelectron spectroscopy (XPS, PHI GENESIS 500, Al Kα X-ray source).

**Figure S5.** XPS survey spectrum of the FN-PAF surface, with the identified elements and their atomic concentrations labeled.

The corrosion behavior of FN-PAF was assessed by potentiodynamic polarization using an electrochemical workstation (Gamry Reference 600). A three-electrode cell filled with 3.5 wt.% NaCl electrolyte was used, with graphite and Ag/AgCl (saturated KCl) as the counter and reference electrodes. Potentials are reported versus the standard hydrogen electrode (SHE), obtained by adding +0.197 V to the values measured against the Ag/AgCl reference. The working electrode was a parallel bundle of FN-PAFs with both ends sealed in epoxy resin, exposing a defined length of fibers whose total lateral surface area defined the effective electrode area.

**Figure S6.** Potentiodynamic polarization curve of FN-PAF coating in 3.5 wt.% NaCl.

The complex permeability (*μ*_r_ *= μ′* ‒ *j μ″*) of the Fe_0.64_Ni_0.36_ coating was characterized over two frequency ranges. FN-PAFs were cut into 1 mm short fibers and dispersed in molten paraffin at a mass loading of 40 wt.%. The composite was pressed into ring samples. The magnetic ring method (1−200 MHz) used a precision impedance analyzer (Keysight E4991B) with rings of 20 mm outer and 12 mm inner diameter; the coaxial transmission-line method (0.2−18 GHz) used a vector network analyzer (Anritsu MS46322B) with rings of 7.0 mm outer and 3.04 mm inner diameter.

**Figure S7.** Complex permeability of the Fe_0.64_Ni_0.34_ coating, characterized by the magnetic ring method (1−200 MHz) and the coaxial transmission-line method (0.2−18 GHz).

**Section 2. Synthesis of CNT-coated aramid fibrils**

Poly (diallyl dimethyl ammonium chloride) (PDDA, Mw = 400,000 ‒ 500,000) was purchased from Macklin. Poly (sodium 4-styrenesulfonate) (PSS, Mw = 70,000) was purchased from Sigma-Aldrich. Sodium chloride (NaCl, analytical reagent grade, purity ≥ 99%) and sodium hydroxide (NaOH, analytical reagent grade, purity ≥ 85%) were purchased from Damao Chemical Reagent Factory. Carboxylated single-wall carbon nanotubes (SWCNTs, diameter 1‒2 nm, and length distribution 5‒30 μm) were purchased from Chengdu Organic Chemicals. Chopped meta-aramid fibers (AFs, average diameter 14 μm, and length 6 mm), and meta-aramid fibrils (AFBs) were provided by Yantai Tayho Advanced Materials Co. LTD.

The polyelectrolyte multilayer technique was used to assemble SWCNTs onto the AFB surface ^[2]^. Polyelectrolyte solutions of PDDA (1 g·L⁻^1^) and PSS (1 g·L⁻^1^) were prepared in 0.5 mol·L⁻^1^ NaCl solution, with the pH adjusted to 10 using NaOH. For each deposition, 2 g of AFBs was added to 1 L of the respective polyelectrolyte solution and dispersed into a slurry with a blade stirrer (Kormes BP-300) at 7000 rpm, promoting electrostatic deposition of the polymer onto the AFB surface. The deposition followed the sequence PDDA → PSS → PDDA; this tri-layer sequence provides a uniform, positively charged surface that promotes adhesion of the subsequent SWCNT layer. The functionalized AFBs were then immersed in 1 L of SWCNT dispersion (0.1 g·L^‒1^ carboxylated SWCNTs in 0.5 mol·L⁻^1^ NaCl, pH 10). Driven by electrostatic attraction between the negatively charged carboxyl groups on the SWCNTs and the positively charged outer PDDA layer, the SWCNTs were uniformly adsorbed to form the CNT-coated AFBs (CAFBs).

**Section 3. Wet-laid papermaking**

The papermaking process was adapted from established protocols ^[3]^. FN-PAF filaments were cut into 6 mm segments. FN-PAFs and AFs served as the structural fibers, while CAFBs and AFBs functioned as the fibrillar binders, with a fiber-to-fibril volume ratio of 6:4 was maintained across all variants. These components were dispersed in 5 L of deionized water with a fiber dissociation disperser (Disper 01B) at 5000 rpm for 20 minutes. The suspension was vacuum-dewatered on a standard handsheet former (Sheet Former 200GS-T) to form a cohesive wet web, which was then interleaved between two circular absorbent papers (Φ = 200 mm) and vacuum-pressed. The sheet was dried at 100 °C for 2 minutes, yielding magnetic aramid-based papers (MAPs) with a nominal diameter of 200 mm and a mean thickness of 0.25 mm.

Magnetic functionality was introduced by incorporating FN-PAFs, used either alone or blended with meta-aramid fibers (AFs); the volumetric faction of FN-PAFs within the total fiber content (*Φ*_FN-PAF_) served as the primary compositional variable. Two types of fibrils with contrasting electrical properties were used: insulating AFBs (resistivity ≈ 10^12^ Ω∙m) and conductive CAFBs (resistivity ≈ 10^5^ Ω∙m) ^[2]^. The volumetric compositions of the twelve papers are tabulated in **Table S1**.

**Table S1.** Volumetric fractions of the fibers (AF and FN-PAF) and fibrils (AFB and CAFB) in the twelve paper formulations.

| *Magnetic aramid-based papers*  *Fibers + Fibrils = 100 vol.%* | | Fibers (60 vol.%): *Φ*_FN-PAF_ | | | | | |
| --- | --- | --- | --- | --- | --- | --- | --- |
|  |  | 0% | 5% | 10% | 20% | 30% | 50% |
| Fibrils  (40 vol.%) | 0% CAFB | AP | MAP-5 | MAP-10 | MAP-20 | MAP-30 | MAP-50 |
|  | 20% CAFB | CAP | CMAP-5 | CMAP-10 | CMAP-20 | CMAP-30 | CMAP-50 |

* *Φ*_FN-PAF_ denotes the volume fraction of FN-PAFs within the total fiber content.

To establish comprehensive fitting curves for the MAPs, auxiliary samples with *Φ*_FN-PAF_ of 15, 70, and 100 vol.% were prepared. The surface (*ρ*_s_) and bulk (*ρ*_v_) resistivity of the pristine aramid papers (APs) were 1.3 × 10^15^ Ω·sq^‒1^ and 2.8 × 10^14^ Ω·m, respectively. At *Φ*_FN-PAF_ =100 vol.%, *ρ*_s_ and *ρ*_v_ decreased to 6.2 × 10^5^ Ω·sq^‒1^ and 1.8 × 10^4^ Ω·m, respectively. The dependence of *ρ*_s_ and *ρ*_v_ on *Φ*_FN-PAF_ was modeled using the McLachlan generalized effective-medium (GEM) theory, as expressed in equations S2–S4 ^[4]^:

$(1-\Phi)\frac{\sigma_{m}^{1/s}-\sigma^{1/s}}{\sigma_{m}^{1/s}+A\sigma^{1/s}}+\Phi\frac{\sigma_{i}^{1/t}-\sigma^{1/t}}{\sigma_{i}^{1/t}+A\sigma^{1/t}}=0$ (S2)

$(1-\Phi)\frac{\sigma_{m}^{-1/s}-\sigma^{-1/s}}{\sigma_{m}^{-1/s}+A\sigma^{-1/s}}+\Phi\frac{\sigma_{i}^{-1/t}-\sigma^{-1/t}}{\sigma_{i}^{-1/t}+A\sigma^{-1/t}}=0$ (S3)

$A=\frac{1-\Phi_{c}}{\Phi_{c}}$ (S4)

where *ρ*, *ρ*_m_, and *ρ*_i_ are the resistivities of the composite, the insulating phase, and the conductive inclusions, respectively. *Φ* denotes the volume fraction of the conductive filler; *Φ*_c_ is the percolation threshold; and *s* and *t* are critical exponents. To simplify the fitting, we assumed *s* = *t*, corresponding to a symmetric resistivity transition about the percolation threshold. Because the fiber fraction in all MAPs is fixed at 60 vol.%, and *Φ*_FN-PAF_ is the volume fraction of FN-PAFs within the total fiber content, the conductive-filler fraction was taken as *Φ* = 0.6 × *Φ*_FN-PAF_. The fitted parameters are summarized in Table S2.

**Table S2.** Fitted parameters (ρ_m_, ρ_i_, *Φ*_c_, and s) for the surface (*ρ*_s_) and bulk (*ρ*_v_) resistivity models of the MAPs.

| *Type of resistivity* | *ρ*_m_ | *ρ*_i_ | *Φ*_c_ | s |
| --- | --- | --- | --- | --- |
| Surface resistivity (*ρ*_s_) | 1.1 × 10^15^ Ω·sq^‒1^ | 2.0 × 10^5^ Ω·sq^‒1^ | 41.8 vol.% | 2.3 |
| Bulk resistivity (*ρ*_v_) | 3.2 × 10^14^ Ω·m | 7.0 × 10^3^ Ω·m | 31.3 vol.% | 1.4 |

**Figure S8.** Normalized magnetic hysteresis loops of (a) MAP-50 and (b) CMAP-50, measured with the magnetic field applied parallel (in-plane) and perpendicular (out-of-plane) to the paper surface. The insets show magnified views of the low field region (−18 to +18 Oe).

**Figure S9.** Tensile properties of the twelve paper formulations: (a) stress-strain curves, (b) failure strain, (c) tensile strength, and (d) elastic modulus.

**Section 4. Electromagnetic properties**

The complex permittivity and permeability were characterized by the coaxial transmission-line method over 1‒18 GHz ^[5]^. To meet the sample-thickness requirement, measurements were performed on stacked paper sheets (Figure S10): three AP layers and two MAP layers were alternately stacked and consolidated with a paraffin adhesive. This alternating architecture provides the thickness required for coaxial measurement while preventing a continuous conductive fiber network from forming across the sample. Such isolation is essential to suppress interlayer shielding, which would otherwise distort the extracted intrinsic material parameters.

**Figure S10.** Coaxial S-parameter characterization of the magnetic aramid-based papers. (a) Schematic of the S-parameter measurement on the stacked sample, and (b) photograph of a representative specimen showing the alternating AP/MAP stack.

**Figure S11.** Complex permittivity (*ε′* and *ε″*) of the AP and AP/paraffin specimens measured over the X-band (8−12 GHz).

The AP/paraffin specimen exhibited a real permittivity (*ε′*) of 2.5 across the X-band, whereas standalone AP (without paraffin) gave *ε′ ≈* 1.2 (Figure S11). Because aramid-based matrices show relatively stable dielectric properties over the investigated frequency range, the difference of ~1.3‒attributed to the paraffin binder‒was treated as a constant offset and subtracted from the measured *ε′* to recover the intrinsic permittivity of the aramid papers.

The attenuation constant (α) quantifies the intrinsic dissipation capability of the material and is given by equation S5 ^[6]^:

$\alpha=\frac{\sqrt{2}\pi f}{c}\sqrt{(\mu''\varepsilon''-\mu'\varepsilon')+\sqrt{(\mu''\varepsilon''-\mu'\varepsilon')^{2}+(\mu'\varepsilon''+\mu''\varepsilon')^{2}}}$ (S5)

where *c* is the speed of light in free space. A higher *α* value indicates stronger attenuation of electromagnetic waves within the material.

The eddy-current loss coefficient (*C*_0_), which comprises inter-particle (*C*_inter_) and intra-particle (*C*_intra_) contributions, is given by equation S6 ^[7]^:

$C_{0}=\mu''(\mu')^{-2}f^{-1}$ (S6)

The microwave absorption performance was evaluated through the frequency-dependent reflection loss (*RL*), calculated with the Dallenbach model. This model treats a single-layer absorber backed by a perfect electric conductor (PEC). From transmission-line theory, the normalized input impedance (*Z*_in_) and *RL* are given by equation S6 and S7 ^[8]^:

$Z_{in}=Z_{0}\sqrt{\frac{\mu_{r}}{\varepsilon_{r}}}\tanh\left[j(\frac{2\pi fd}{c})\sqrt{\mu_{r}\varepsilon_{r}}\right]$ (S6)

$RL=20\log_{10}\left|\frac{Z_{in}-Z_{0}}{Z_{in}+Z_{0}}\right|$ (S7)

where *Z*_0_ is the impedance of free space, *d* is the thickness of the single-layer absorber, and *c* is the speed of light. The measured electromagnetic parameters were used to calculate *RL* for thicknesses from 0.1 to 5.0 mm over 1–18 GHz. Effective absorption also requires good impedance matching, which allows incident waves to enter the material rather than reflect at the surface. Ideally, the input impedance of the absorber equals that of free space. Accordingly, the degree of impedance matching was assessed from the modulus of the normalized input impedance, |Z_in_ / Z_0_|. A value of |Z_in_ / Z_0_| approaching unity indicates good impedance matching, and minimizing surface reflection.

Finite-element simulations were performed with the frequency-domain solver in CST Studio Suite to evaluate the electric field (***E***) and power-loss density (*P*_loss_) distributions within the magnetic aramid-based papers. Unit-cell models (20 × 20 × 4.5 mm^3^) with periodic boundary conditions in the *xy*-plane were used to represent an infinite periodic array under normally incident radiation. Field distributions were analyzed at 2.7 GHz. At the PEC boundary, the tangential electric field vanishes (0 V∙m^‒1^) at the sample-PEC interface. The electric field at the incident (upper) surface is governed by the effective wavelength in the material, $\lambda_{m}=c/(f\sqrt{\mu_{r}\varepsilon_{r}})$; samples with shorter internal wavelengths therefore exhibit higher surface field intensities.

**Figure S12.** Frequency-dependent electromagnetic parameters of the MPA variants: (a) complex permittivity (*ε*′ and *ε*″), (b) complex permeability (*μ*′ and *μ*″), and (c) the dielectric and magnetic loss tangents (tanδ_ε_ and tanδ_μ_).

**Figure S13.** Frequency dependence of the eddy-current loss coefficient (*C*_0_) for the MPA variants.

**Figure S14.** (a‒e) Reflection loss maps and (f‒j) relative input impedance maps for the MAPs (*Φ*_FN-PAF_ = 5−50 vol.%) over 1‒8 GHz.

**Figure S15.** (a) Electric field and (b) power-loss density for two representative MAP variants (MAP-20 and MAP-30; *d* = 4.5 mm) under normal incidence at 2.7 GHz.

**Figure S17.** Frequency dependence of the eddy-current loss coefficient (*C*_0_) for the CMPA variants.

**Figure S16.** Frequency-dependent electromagnetic parameters of the CMPA variants: (a) complex permittivity (*ε*′ and *ε*″), (b) complex permeability (*μ*′ and *μ*″), and (c) the dielectric and magnetic loss tangents (tanδ_ε_ and tanδ_μ_).

**Figure S18.** (a‒e) Reflection loss maps and (f‒j) relative input impedance maps for the CMAPs (*Φ*_FN-PAF_ = 5−50 vol.%) over 1‒8 GHz.

**Figure S19.** (a) Electric field and (b) power-loss density for two representative CMAP variants (CMAP-20 and CMAP-30; *d* = 4.5 mm) under normal incidence at 2.7 GHz.

**Section 5. Free-space reflectance measurements**

To validate the practical microwave absorption performance of the composite papers, free-space reflectance measurements were performed using the Naval Research Laboratory (NRL) arch method. Specimens (180 mm × 180 mm^2^) were mounted on a metallic backing plate to provide a perfect-electric-conductor (PEC) boundary condition. The assembly was illuminated at normal incidence by a pair of wideband horn antennas connected to a vector network analyzer (VNA) over 1‒18 GHz. Because the single-layer thickness of the papers is uniform (~0.25 mm), absorbers of prescribed thickness were built by layer-by-layer stacking, allowing the total thickness to be tuned in 0.25 mm increments.

To benchmark the S-band performance of CMAP-20, it was compared against five representative FeNi-based composites with distinct morphologies and architectures, using a five-dimensional scoring framework comprising filler loading, matching thickness, *EAB*, *μ′* at 3 GHz, and *RL_max_* frequency. Each parameter was normalized on a 0‒5 scale according to the criteria in Table S3, and results were visualized via a spider chart (Figure 5c).

**Figure S20.** Experimental free-space reflectance spectra of the CMAP-20 over 1–18 GHz.

| *Scores* | Filler Loading (wt. %) | Thickness (mm) | EAB (GHz) | *μʹ* @ 3 GHz | RL_max_ Frequency (GHz) |
| --- | --- | --- | --- | --- | --- |
| 0 | N.A. | N.A. | N.A. | N.A. | N.A. |
| 1 | > 66 | > 5.0 | < 0.65 | < 1.1 | > 4.0 |
| 2 | 52 ~ 66 | 4.2 ~ 5.0 | 0.65 ~ 0.8 | 1.1 ~ 1.3 | 3.5 ~ 4.0 |
| 3 | 38 ~ 52 | 3.4 ~ 4.2 | 0.8 ~ 0.95 | 1.3 ~ 1.5 | 3.0 ~ 3.5 |
| 4 | 24 ~ 38 | 2.6 ~ 3.4 | 0.95 ~ 1.1 | 1.5 ~ 1.7 | 2.5 ~ 3.0 |
| 5 | ≤ 24 | ≤ 2.6 | ≥ 1.1 | ≥ 1.7 | ≤ 2.5 |

**Table S3.** Quantitative criteria for scoring the S-band microwave absorption performance of the FeNi-based composites (radar chart, Figure 5c).

| *Sample* | AP | CMAP-5 | CMAP-10 | **CMAP-20** | CMAP-30 | CMAP-50 |
| --- | --- | --- | --- | --- | --- | --- |
| Mass fraction of magnetic constituents (wt.%) | 0 | 6.2 | 11.7 | **21.3** | 29.4 | 42.0 |
| Grammage of paper (g·m^‒2^) | 60.0 | 63.2 | 66.5 | **73.0** | 79.6 | 92.6 |

**Table S4.** Physical and structural specifications of the CMAPs at different *Φ*_FN-PAF_ values.

**Table S5**. Comparison of the S-band microwave absorption performance of representative FeNi-based composites across the five evaluation criteria.

| *Parameters* | Micro-spheres ^[9]^ | Nano-wire ^[10]^ | Flakes ^[11]^ | CF@FeNi ^[12]^ | CNT/FeNi ^[13]^ | **This work** |
| --- | --- | --- | --- | --- | --- | --- |
| Filler Loading (wt.%) | 86 | 25 | 80 | 23 | 40 | **22** |
| Thickness (mm) | 4.0 | 3.5 | 2.4 | 5.0 | 5.2 | **3.3** |
| EAB (GHz) | 0.9 | 0.7 | 1.2 | 0.6 | 0.9 | **1.0** |
| *μʹ* @ 3 GHz | 1.49 | 1.18 | 2.92 | 1.02 | 1.33 | **1.32** |
| RL_max_ Frequency (GHz) | 2.82 | 2.77 | 3.14 | 3.81 | 3.41 | **3.43** |

* CF: carbon fiber

**Section 6. Mechanism analysis**

The complex permeability (*μ*_r_ *= μ′* ‒ *j μ″*) of the MAP series was characterized over 0.01–18 GHz using two complementary methods. The magnetic ring method was used over 0.01–0.1 GHz, and the coaxial transmission-line method over 0.1–18 GHz. For the magnetic ring method, twenty annular rings (outer diameter: 20 mm; inner diameter: 12 mm) were cut from the MAP samples and stacked in a dedicated ring mold. From the measured inductance and quality factor of the stacked rings, the complex permeability was calculated using equations S8 and S9 ^[14]^:

$\mu'=\frac{(L_{s}-L_{0})l}{\mu_{0}N^{2}A}+1$ (S8)

$\mu''=\frac{\mu'}{Q_{s}}-\frac{1}{Q_{0}}$ (S9)

where *L*_s_ and *Q*_s_ are the inductance and quality factor of the sample-loaded coil, and *L*_0_ and *Q*_0_ are those of the empty coil. *N* is the number of winding turns, *A* is the effective cross-sectional area of the ring mold, *l* is the effective magnetic path length, and *μ*_0_ is the permeability of free space.

**Figure S21.** Complex permeability spectra (*μ*′, *μ*″) for the MAP series measured in the low-frequency regimes: (a) 0.01‒0.1 GHz (magnetic ring method), and (b) 0.1‒1.0 GHz (coaxial line method).

Experimental free-space reflectance spectra of the CMAP-20 measured across the 1–18 GHz frequency range.

The complex magnetic susceptibility is defined as *χ* = *μ*_r_ ‒ 1. Based on ferromagnetic resonance (FMR) theory and the Landau-Lifshitz-Gilbert (LLG) equation, the frequency dispersion of the complex susceptibility, governed by the magnetic anisotropy field, is given by equation S10 ^[15]^:

$\chi(\omega)=\frac{\chi_{DC}\left[1+j\frac{\alpha\omega}{\gamma H_{k}+(N_{y}-N_{Z})\omega_{m}}\right]}{1-\frac{\omega^{2}}{\omega_{r}^{2}}+j(\frac{2\alpha\omega}{\omega_{r}^{2}})\left[\gamma H_{k}+\frac{1}{2}(N_{x}-N_{y}-2N_{Z})\omega_{m}\right]}$ (S10)

where *ω*_m_=*γ*2π*M*_s_, *χ*_DC_ is the static susceptibility, *γ* is the gyromagnetic ratio, *α* is the Gilbert damping factor, *H*_k_ is the magnetocrystalline anisotropy field, *ω*_r_ is the resonant frequency, and *N*_x_, *N*_y_, and *N*_z_ are the demagnetizing factors along the *x*, *y*, and *z* axes.

A simplified dispersion function was used for the fitting (equation S11):

$\mu_{r}(\omega)=\chi(\omega)+1=\frac{A(1+j\frac{\omega}{B})}{1-\frac{\omega^{2}}{C^{2}}+j(\frac{2\omega}{D})}$ (S11)

where A, B, C, and D are independent fitting constants related to the static susceptibility and resonance parameters. Fitting the *μ′* data of MAP-50 over 0.01−10 GHz yielded the complex permeability dispersion curve (Figure 5e), which shows a broad ferromagnetic resonance peak spanning about three orders of magnitude in frequency, with a fitted resonance frequency of 0.087 GHz.

Beyond magnetic loss, the dielectric response is affected by the antenna resonance of the high-aspect-ratio fibers. The fundamental resonance frequency (*f*_r_) of the conducting fibers was estimated using the dipole-antenna approximation ^[16]^, with the antenna resonance following the Lorentz dispersion model (equation S12) ^[17]^:

$\varepsilon_{r}(\omega)=\varepsilon_{\infty}+\frac{\omega_{P}^{2}}{\omega_{r}^{2}-\omega^{2}+j\omega\Gamma_{0}}$ (S12)

where *ω*_P_ is the plasma frequency, *ω*_r_ is the angular resonance frequency, and *Γ*_0_ is the damping constant. The calculated antenna resonance frequency of 12.5 GHz agrees closely with the measured resonance at 11.8 GHz (Figure S12a and S16a). Located in the 6‒18 GHz range, the resonance enhances the dielectric loss and broadens the absorption bandwidth at high frequencies.

**References**

[1]	a)T. Jariyanorawiss, C. Thumkanon, presented at *2018 IEEE International WIE Conference on Electrical and Computer Engineering (WIECON-ECE)*, 14-16 December, **2018**; b)G. S. Smith, *European Journal of Physics* **2014**, 35, 025002.

[2]	Y. Wu, L. Chen, Y. Han, P. Liu, H. Xu, G. Yu, Y. Wang, T. Wen, W. Ju, J. Gu, *Nano Research* **2023**, 16, 7801.

[3]	H. Wang, X. Xiu, Y. Wang, Q. Xue, W. Ju, W. Che, S. Liao, H. Jiang, M. Tang, J. Long, J. Hu, *Composites Part B: Engineering* **2020**, 202, 108378.

[4]	X. Song, J. Ji, N. Zhou, M. Chen, R. Qu, H. Li, L. a. Zhang, S. Ma, Z. Ma, Y. Wei, *Progress in Materials Science* **2024**, 144, 101288.

[5]	a)E. Mattei, S. E. Lauro, E. Pettinelli, G. Vannaroni, *IEEE Transactions on Instrumentation and Measurement* **2013**, 62, 2938; b)Y. Han, H. Guo, H. Qiu, J. Hu, M. He, X. Shi, Y. Zhang, J. Kong, J. Gu, *Advanced Functional Materials* **2025**, 35, 2506803.

[6]	S. Gao, G. Zhang, Y. Wang, X. Han, Y. Huang, P. Liu, *Journal of Materials Science & Technology* **2021**, 88, 56.

[7]	M. Wu, Y. D. Zhang, S. Hui, T. D. Xiao, S. Ge, W. A. Hines, J. I. Budnick, G. W. Taylor, *Applied Physics Letters* **2002**, 80, 4404.

[8]	Y. Liu, K. Zhao, M. G. B. Drew, Y. Liu, *AIP Advances* **2018**, 8, 015223.

[9]	Y. Feng, T. Qiu, *Journal of Alloys and Compounds* **2012**, 513, 455.

[10]	R. Cai, P.-A. Yang, H. Ruan, X. Huang, Z. Zhou, Q. Chen, Y. Zhang, X. Gong, Y. Gui, R. Li, *Applied Surface Science* **2024**, 668, 160434.

[11]	W. Li, J. Lv, X. Zhou, J. Zheng, Y. Ying, L. Qiao, J. Yu, S. Che, *Journal of Magnetism and Magnetic Materials* **2017**, 426, 504.

[12]	C. Wang, H. Jiang, X. Cao, X. He, X. Chen, B. Cui, X. Xu, Y. Wang, C. Wang, *Advanced Fiber Materials* **2024**, 6, 1640.

[13]	F. Wen, F. Zhang, J. Xiang, W. Hu, S. Yuan, Z. Liu, *Journal of Magnetism and Magnetic Materials* **2013**, 343, 281.

[14]	Z. Guo, J. Wang, W. Chen, D. Chen, H. Sun, Z. Xue, C. Wang, *Materials & Design* **2020**, 192, 108769.

[15]	a)O. Reynet, A.-L. Adenot, S. Deprot, O. Acher, M. Latrach, *Physical Review B* **2002**, 66, 094412; b)R. Moore, *Electromagnetic Composites Handbook, Second Edition*, McGraw-Hill Education, New York, N.Y **2016**.

[16]	A. N. Lagarkov, A. K. Sarychev, *Physical Review B* **1996**, 53, 6318.

[17]	F. Qin, M. Peng, D. Estevez, C. Brosseau, *Journal of Applied Physics* **2022**, 132, 101101.
